# Maternal Antibiotic Exposure and the Risk of Developing Antenatal Depressive Symptoms

**DOI:** 10.3390/jcm13051434

**Published:** 2024-03-01

**Authors:** Mahsa Pouranayatihosseinabad, Maggie Taylor, Jason A. Hawrelak, Gregory M. Peterson, Felicity Veal, Tristan Ling, Mackenzie Williams, Megan Whatley, Kyan Ahdieh, Corinne Mirkazemi

**Affiliations:** 1 School of Pharmacy and Pharmacology, College of Health and Medicine, University of Tasmania, Hobart, TAS 7005, Australiag.peterson@utas.edu.au (G.M.P.); corinne.mirkazemi@utas.edu.au (C.M.); 2 Human Nutrition and Functional Medicine, University of Western States, Portland, OR 97213, USA; 3 Department of Obstetrics and Gynaecology, Mater Mothers’ Hospital, Brisbane, QLD 4101, Australia; 4 Launceston Medical Centre, Health Hub, Launceston, TAS 7250, Australia

**Keywords:** care, antenatal, depressive disorder, depression, antibiotics, pregnancy, risk factors

## Abstract

**Background:** Antenatal depression is common and has significant consequences. The literature suggests that antibiotic exposure may be associated with depression. Many individuals are exposed to antibiotics during pregnancy. Further investigation of the association between antenatal antibiotic use and the development of depression during pregnancy is needed. **Methods:** A national prospective observational cohort study of pregnant individuals was undertaken using an online survey, completed during the third trimester. Antenatal depressive symptoms (ADSs) were defined as having an Edinburgh Postnatal Depression Scale score of ≥13 and/or receiving a clinical diagnosis of depression. **Results:** One in six individuals (16.5%, *n* = 977) experienced ADSs during their pregnancy, of whom 37.9% received a depression diagnosis. There was no relationship between antibiotic use and the development of ADSs. Four factors were identified as significant independent predictors of ADSs: personal history of depression, severe nausea and vomiting causing an inability to eat, emotional abuse from an intimate partner within the prior 12 months, and not having a university degree. **Conclusions:** Antenatal antibiotic use was not associated with the development of ADSs. Given the high incidence of undiagnosed depression, new strategies and models of care that prioritise individuals with risk factors may be required to optimise antenatal care.

## 1. Introduction

Depression is a debilitating health condition affecting 322 million individuals across the world, with a higher prevalence in females compared to males (5.1% vs. 3.6%) [1]. Depressive symptoms are commonly experienced during pregnancy, with up to one out of five pregnant individuals experiencing them during the antenatal period [2]. Notably, the rate at which individuals experience depressive symptoms during the first trimester is similar to that of non-pregnant females; however, the rate at which they experience depressive symptoms during the second and third trimesters is nearly twice as high compared to non-pregnant females [3].

The consequences of antenatal depressive symptoms (ADSs) can be significant for both gestational parents and their babies. For example, experiencing ADSs is associated with increased gestational parent morbidity, including perinatal complications and an increased incidence of operative delivery [4]. There is some evidence to suggest individuals affected by ADSs are also at an increased risk of preterm delivery [5,6] and that their babies are at an increased risk of low birth weight [5,7]. Moreover, babies born to gestational parents who suffer from depressive symptoms during pregnancy may experience decreased breastfeeding initiation [6] and lower cognitive development [8]. Experiencing ADSs is also one of the main risk factors for experiencing postpartum depressive symptoms [9], which can itself have a negative impact on gestational parent–infant bonding and infant development [10].

A range of different risk factors have been identified for developing ADSs, including sociodemographic, psychological, and biological factors [9,11]. One potential risk factor that has yet to be explored in depth is exposure to antibiotics during pregnancy. Up to 33% of individuals are prescribed oral antibiotics during pregnancy and one-third of these receive more than one antibiotic prescription [12]. Most commonly, they are prescribed oral antibiotics for the treatment of skin, urinary, respiratory, and ear infections, although prescribing antibiotics without a clear clinical indication is not uncommon during pregnancy [12]. The gastrointestinal microbiota ecosystem, which is believed to contribute to the regulation of mood and emotion [13], is altered by antibiotics [14], including during pregnancy [15]. Prior studies have identified a potential link between antibiotic exposure and the development of depression [16], including one study by Field et al. [17] that explored it as a risk factor for antenatal depression. Notably though, there are many significant limitations associated with the studies published to date [16], including, in the case of the study by Field et al. [17], failure to control for many previously reported risk factors for the development of ADSs, including history of depression [2,11]. Recognition of the dearth of quality literature exploring this potential association prompted the design of the current study.

The aim and primary objective of this study was to investigate the association between antenatal antibiotic exposure and the development of depressive symptoms during pregnancy, considering known risk factors. Its secondary objectives included estimating the current point incidence of ADSs in Australia and associated factors. We hypothesised that antenatal antibiotic exposure would be associated with the development of ADSs.

## 2. Materials and Methods

### 2.1. Design and Study Population

The data reported in this paper are from an ongoing Australia-wide prospective longitudinal observational cohort study, called “*The Maternal Experience Study: Factors affecting your health and wellbeing following the birth of your child*”, and detailed information on the methodology has been previously reported [18]. In brief, the study includes four online surveys to be completed over the course of each participant’s pregnancy and postpartum journey. The first is completed during the second half of the third trimester of pregnancy (from approximately 32 weeks’ gestation), and the other three at approximately 6 weeks, 6 months, and 12 months postpartum. This paper reports on the results of the antenatal survey, noting that the original primary focus of the research was postpartum depressive symptoms and the data collection for that phase is continuing at the time of writing.

Voluntary response sampling was employed in this study. This study was promoted through various methods, including online platforms (such as posts in pregnancy-related Facebook groups, e.g., Pregnancy Support Group Australia) and targeted paid advertising (through Facebook, Google, and Instagram), as well as by distributing study flyers at obstetric, radiology, and midwifery clinics, supermarkets, and pregnancy-associated service areas (e.g., studios specialising in yoga in pregnancy). This mixed approach to recruitment was utilised to minimise selection bias; however, it made the response rate impossible to calculate. Prize draws, including 41 chances to win a gift voucher for completing the study, were used to encourage participation. Pregnant individuals were recruited between August 2021 and June 2022, and could consent to participate at any time during their index pregnancy. They received the antenatal survey no sooner than 32 weeks gestation (August 2021–November 2022). If they did not complete the survey, two reminder notices were sent to them approximately two weeks apart. To better capture and reflect the impact of exposure to antibiotics during pregnancy and the development of antenatal depression, the depression screening was conducted no sooner than 32 weeks gestation. Furthermore, choosing this time frame would also allow for more consistency and better control for the gathered data among pregnant individuals, whose obstetric, physical, and psychological conditions can vary between different trimesters; a later time frame was not utilised due to the increased likelihood of losing recruited participants to preterm labour. Questions pertaining specifically to antenatal depression were only included after recruitment had been active for several months. Pregnant individuals residing in Australia and aged at least 18 years were eligible. Participants were excluded prospectively from the study if they were a surrogate. Retrospective exclusion criteria relevant for this analysis included having a stillbirth or miscarriage prior to completing the survey. Ethics approval for this study was obtained from the University of Tasmania Human Research Ethics Committee (H0021790).

### 2.2. Survey Variables and Outcome Measure

Where possible, validated scales or previously used questionnaires were used [18], including the Edinburgh Postnatal Depression Scale (EPDS) [19]. The EPDS is a 10-item scale designed to identify participants experiencing depressive symptoms in the postpartum period; however, it has also been validated for use in pregnancy [20]. An EPDS score of or above 13, the standard cut-off score recommended when screening for probable major depression, has been used in studies conducted in Australia and internationally [19,21,22,23,24,25]. Moreover, the Royal Australian and New Zealand College of Obstetricians and Gynaecologists (RANZCOG) recommends that individuals who score 13 or above be further reviewed, as this score may suggest a crisis [26]. The primary outcome (depressive symptoms) was defined as either having an EPDS score of at least 13 during their antenatal survey and/or receiving a diagnosis of depression made or confirmed by a medical practitioner (e.g., general practitioner/family doctor, psychiatrist, obstetrician) during their index pregnancy.

As part of the data collection process, individuals were also asked whether they had taken any courses of antibiotics during their pregnancy. If they answered yes, they were asked to provide more details about the name of the antibiotic, the dose, the number of courses, the duration of therapy, and in which trimester they had taken them. To reduce the potential for illnesses requiring antibiotics to themselves bias the results, individuals were asked prior to commencing the survey whether they were using or had finished a course of antibiotics within the prior 14 days. When they had, they were not able to commence the survey until at least 14 days had passed following the self-reported expected date of completion for their antibiotic course. This allowed for recovery in the first week following antibiotics, and then assessment of their mental health state at the end of the second week (noting that the EPDS asks individuals to respond reflecting on their experiences in the prior 7 days).

### 2.3. Statistical Analysis

A browser-based metadata-driven capture system, Research Electronic Data Capture (REDCap) [27], was used for survey design and data collection. The collected data were then transferred to a Microsoft Excel document for data cleaning (including range and logic checks). From there, the de-identified quantitative data were analysed using version 27.0 of SPSS (Statistical Package for Social Sciences, IBM^®^ Armonk, New York, NY, USA). Quantitative data were gathered using previously validated scales and tools (for a full description, see protocol study [18]). Many of these, including variables gathering information pertaining to symptoms of depression, stress, and anxiety, and those exploring quality of sleep, level of social support, and whether abuse from an intimate partner had been experienced in the prior 12 months, were analysed using pre-defined category boundaries. Other variables, such as participation in physical activity and antibiotic use, were analysed using dichotomous categories (yes/no). Most quantitative data were summarised using frequencies and percentages; for participant age and gestational age, means with standard deviations were utilised. A series of bivariate analyses followed by a multiple logistic regression analysis were conducted. The logistic regression used the default listwise deletion of missing data. Variables with *p* < 0.01 in the bivariate analyses were subsequently checked for multicollinearity and interdependence (where *p* < 0.001 was deemed to be significant) before being included in the multiple logistic regression examining associations with depression. A sample size calculation was conducted (using Peduzzi’s formula [28]) assuming ten independent variables in the logistic regression, and an anticipated ADS rate of 5.9% [29]. The target sample size was set at 2203 accounting for a projected 30% drop out. Only *p* < 0.05 in the multiple logistic regression was considered statistically significant.

We acknowledge that collecting data at a discrete time point (i.e., just during the third trimester) creates some uncertainty regarding the potential relationship between the timing of antibiotic use and the timing of depressive symptoms. To address this issue, a sensitivity analysis was also conducted using only an EPDS score of at least 13 as the outcome measure, as the timing of the administration of the EPDS tool is clear in relation to any antibiotic use that is disclosed in the same survey.

## 3. Results

### 3.1. Demographic Information and Participant Characteristics

One thousand five hundred eighty-three individuals consented to participate. Of these, 977 individuals completed the survey questions specifically relating to antenatal depression. They completed the survey at some point between 31.9 and 42.0 gestation weeks (mean of 33.4 weeks’ gestation (standard deviation (SD) = 2.1)). The majority of these individuals were tertiary educated, typically living with a partner, and born in Australia (Table 1). Participant age ranged from 18 to 51 years, with a mean age of 32.1 years (SD = 3.9 years). This study recruited individuals from all the states and territories of Australia, with the greatest number of participants residing in New South Wales (232, 23.8%) and Victoria (229, 23.5%), followed by Tasmania (162, 16.6%) and Queensland (159, 16.3%). The vast majority identified themselves as female, with only one individual identifying as non-binary, one participant preferring not to report their gender identity, and one person identifying as both non-binary and female.

More than two in five individuals (43.5%) reported a personal history of any mental health condition, including depression. Antibiotic exposure during pregnancy was reported by almost one in five individuals (Table 2), with most of these individuals reporting using antibiotics during the first or second trimester of their pregnancy (Table S1). Conversely, the consumption of probiotics was greatest during the third trimester (Table S1). More than two-thirds stated that their pregnancy (e.g., appointments, birth plan) had been affected by the COVID-19 pandemic, and over half of the participants reported that the pandemic and its associated social isolation measures had either moderately or highly affected their mental health in a negative way (Table S2).

### 3.2. Incidence of Antenatal Depression Symptoms

Sixty-one individuals reported being diagnosed with depression during their index pregnancy (6.2%), and a further 100 individuals had an EPDS score of at least 13 when completing the survey during their third trimester (10.2%, *n* = 977). The overall point incidence of depressive symptoms was therefore 16.5% during the index pregnancy (161, *n* = 977). Twenty-four individuals who reported being diagnosed with depression (39.3%, *n* = 61) did not have an elevated EPDS score at the time of completing the survey.

### 3.3. Unadjusted Statistical Results

The primary aim was to investigate any association between antibiotic exposure during the index pregnancy and the development of depressive symptoms. A significant association was not found (Table 2). Similarly, the consumption of probiotics during pre-conception and pregnancy did not appear to provide any protection against or show any association with the risk of developing depressive symptoms.

There was a statistically significant inverse association between ADSs and having a fortnightly household income of AUD 2000 or more after paying tax (Table 1) and receiving moderate or high levels of social support (Table 3). Moreover, the risk of ADSs was found to be significantly lower in participants who had a tertiary degree (Table 1), had any sort of paid job (Table 1), and in those who had planned to become pregnant with their index pregnancy (Table 2). Being physically active during pregnancy also appeared to be a protective factor against ADSs (Table 4).

Conversely, cigarette smoking during pregnancy and experiencing vomiting (with or without nausea) to the extent that it significantly impacted the ability to eat were associated with an increased risk of experiencing ADSs (Table 2 and Table 4). There was also a positive association with having a previous personal history of depression, a family history of any mental health condition, and experiencing emotional abuse by an intimate partner in the prior 12 months (Table 3).

### 3.4. Adjusted Statistical Results

In this study, a large range of variables previously identified as risk factors for the development of ADSs were investigated. Variables were carefully selected for the multiple logistic regression to avoid confounding bias. For example, in the process of choosing variables to be included in the multiple logistic regression, some variables pertaining to quality of sleep, stress, and anxiety (Table S2) that were identified in the bivariate analysis as being associated with the primary outcome were subsequently excluded on the basis that they could be interpreted as symptoms of depression. Also, as outlined in Table S3, there were significant co-dependences between some variables. For example, social support was highly associated with six other independent variables (*p* < 0.001), including education. The same applied to planned pregnancy and physical activity. Furthermore, education was considered as a marker of socioeconomic status over income level and employment status, and it was highly related to cigarette smoking. Between personal history of diagnosed depression and family history of any mental health condition (which were highly related to each other), personal history of diagnosed depression was chosen to be incorporated into the logistic regression as (i) participants were deemed to be able to more accurately provide information about their own personal history than their family history and (ii) personal history of depression had been previously recognised as a risk factor for antenatal depression [2,11,30].

Therefore, the multiple logistic regression analysis included four potential factors (Table 5). All these variables remained significantly associated with the risk of developing depressive symptoms. Having a personal history of depression, experiencing vomiting (with or without nausea) to the extent that it significantly impacted the ability to eat, and experiencing emotional abuse from an intimate partner within the prior 12 months were each independently associated with ADSs, while being university-educated appeared to be a protective factor against developing ADSs. To control for the possibility that the influence of other factors may have affected the variable of antibiotic exposure from being significantly associated with depressive symptoms in the bivariate analysis, a sensitivity analysis was conducted by adding it to the multiple logistic regression. This analysis confirmed its lack of association with depressive symptoms (Table 5).

The sensitivity analysis utilising only an EPDS score of 13+ as the outcome measure resulted in only minor differences in the variables that were found to be associated with depressive symptoms. In addition to the variables identified in the main analyses, only age was found to be significantly associated (*p* < 0.01), suggesting that the risk of depressive symptoms declined with increasing age. For the same reasons as previously described, only five significant variables from the bivariate analysis were included in the subsequent multiple logistic regression for the sensitivity analysis. In the logistic regression, age was not found to be a significant factor. Only the four variables identified in the main analysis (history of depression, severe nausea and/or vomiting, experiencing emotional abuse from an intimate partner, and not having a university degree) remained significant in the sensitivity analysis (*p* < 0.05).

## 4. Discussion

To our knowledge, this is the first study to comprehensively examine the relationship between antibiotic exposure during pregnancy and the subsequent development of ADSs. There was no evidence of a significant relationship between antibiotic use and the development of ADSs. The only other prior study investigating this association, conducted in the United States of America (USA), reported that individuals exposed to antibiotics in the first 20 weeks of pregnancy were approximately 50% more likely to be depressed at 20 weeks gestation than individuals who were not exposed to antibiotics [17]. However, it did not include a multivariate analysis and therefore failed to control for many risk factors, including socioeconomic variables. It also predominantly recruited individuals with low to middle socioeconomic status, whereas the present study recruited individuals of predominantly middle to upper socioeconomic status who were highly educated. Notably, the difference in depression incidence was large, with Field et al. [17] reporting 42.0% of individuals being depressed in their cohort, compared with 16.5% in the present study. The differences between the socioeconomic backgrounds are a potential reason for this as a significant association between low socioeconomic status and increased depressive symptoms in late pregnancy has been reported in the USA [31].

Our study’s secondary objective was to estimate the current incidence of ADSs in Australia, and to compare it to the nationally relevant literature at large. The current point incidence of ADSs (i.e., defined as either having an EPDS score of 13+ and/or receiving a clinical diagnosis of depression) was found to be 16.5%. Studies conducted prior to the COVID-19 pandemic had previously reported incidence rates for ADSs (defined as having an EPDS score of at least 13) of 5.9% to 8.9%, with cohorts ranging from 1507 to 53,032 individuals [25,29,32]. These studies all recruited individuals either at antenatal clinics [29,32] or via invitation packages sent by staff from the hospital that individuals had booked to give birth at [25] between 2002 and 2005 [25,32] and between 2014 and 2016 [29]. Only one study [33], which also recruited individuals from antenatal clinics, reported a similar rate of ADSs to this study (15.2%, data collected 2002–2004, *n* = 1578).

Since the onset of the COVID-19 pandemic, several studies have reported on the incidence of ADSs in Australia, each using an EPDS cut-off of 13 in their methodology and using similar recruitment (paid/unpaid social media advertising) and data collection (online survey) methods to the current study. In chronological order (according to their data collection periods), these studies have reported the rates of ADSs to be: 26.5% (*n* = 1219, August 2020–February 2021) [34], 26.7% (*n* = 1607, July 2020–January 2021) [35], and 16.4% (*n* = 269, September 2020–November 2021) [36]. We found 14.0% of individuals had an EPDS score of at least 13 (*n* = 977, August 2021–November 2022). The higher rates of ADSs reported by Lequertier et al. [34] and Davis et al. [35] could potentially be due to the studies being conducted around the time of the initiation of strict social isolation measures and internal and external border closure in Australia [34] and prior to the roll-out of the COVID-19 vaccine [37]. In contrast, more than half of the data collected by Frankham et al. [36], and all of the data for the present study, were collected after vaccine roll-out began in Australia in February 2021, and may account for the decline in ADS incidence.

Although the present study did not identify antibiotic exposure as being a predictor of ADSs, it confirmed the significance of four previously identified independent predictors of ADSs. Personal history of depression was a significant predictor of depressive symptoms during pregnancy. This has been previously identified [38,39,40,41], including by Bisetegn et al. [40] and Biratu et al. [41], with similar odds ratios (adjusted odds ratio (adjOR) = 3.48 (95% CI 1.71–7.06), *p* < 0.01 and adjOR = 2.57 (95% CI 1.48–4.48), *p* < 0.001, respectively). Furthermore, in our study, experiencing emotional abuse from an intimate partner within the prior 12 months was a significant predictor of depressive symptoms during pregnancy. This is in agreement with the findings of a meta-analysis of 70 studies that showed an association between lifetime history of abuse (including emotional abuse) and ADSs [42]. Another predictor of antenatal depression identified in the present study was experiencing severe nausea and vomiting to the extent that it affected ability to eat. Similarly, previous studies have reported that individuals experiencing severe levels of nausea and vomiting or hyperemesis gravidarum have an increased risk of ADSs (odds ratio (OR) = 2.99 (95% CI 1.17–7.64), *p* = 0.022 [43] and OR = 14.4 (95% CI 5.29–39.44), *p* < 0.001 [44]). Similar to our study, Abujilban et al. [45] reported that a high education level was protective against ADSs.

It is important to note that these findings are only significant associations and should not be taken as evidence of causation. However, we consider it prudent for greater effort to be placed on ensuring that individuals are adequately and regularly screened for symptoms of depression during pregnancy, in particular targeting those individuals of a lower socioeconomic status, with a history of abuse or depression, or suffering from severe nausea and vomiting during their pregnancy. The present study indicated that 16.5% of individuals experienced ADSs during pregnancy; however, only just over one-third of these individuals (37.9%) appeared to have received a formal diagnosis. This suggests that the reported depressive symptoms may be temporary, that pregnant individuals are more likely to answer the screening questions honestly when anonymous, and/or that increased screening is needed (given the significant association between antenatal depression and postpartum depression [46]). Healthcare professionals, in particular obstetricians, general practitioners, and midwives, are well placed to create a private, supportive, and non-judgmental environment for screening pregnant individuals for signs and symptoms of depression. For enhanced rates of identification and intervention, screening should be integrated into routine care [30]. At the basic level, this includes utilising standardised questionnaires, such as the EPDS, in prenatal visits. More advanced models include a multidisciplinary approach. The New South Wales Health frameworks “SAFE START Strategic Policy” [47] and “Perinatal Integrated Psychosocial Assessment” [48] are two examples where after screening and identifying depressed pregnant individuals, a triaging multidisciplinary team meets to thoroughly evaluate the situation and engage appropriate services where required. Care, however, must be taken to reduce the risk of overburdening healthcare systems by ensuring screening approaches have both high sensitivity and specificity while remaining practical. In both models mentioned above, in addition to the EPDS, a structured psychosocial questionnaire is used to identify which individuals warrant a referral for further assessment. However, both models only ask about previous personal history of depression and do not screen individuals for the three other significant variables that were confirmed in this study as risk factors for ADSs (i.e., experiencing significant nausea and vomiting, not being tertiary educated, and experiencing emotional abuse from an intimate partner within the prior 12 months). Future research could investigate how the inclusion of these three additional variables to the programs’ screening methods affects their sensitivity, specificity, and feasibility.

### Limitations

One of the limitations of this study is its reliance on individuals to provide accurate data, introducing both potential for cognitive bias and recall bias. To mitigate the cognitive bias risk, participants completed the surveys anonymously, and previously validated tools were utilised within the surveys where possible. Another limitation is that information regarding antibiotic use during pregnancy was collected in the mid to late third trimester, whereas most instances of antibiotic use were reported during the first two trimesters. In the entire cohort, there were only six individuals who reported that they were unsure whether they took any antibiotics during their pregnancy and only two individuals self-identified as having taken an antibiotic when, according to the additional information they provided, they had used an antiviral or antifungal medication instead. It is possible, however, that more individuals incorrectly reported using antibiotics when they were prescribed another anti-infective class, as many participants did not provide any detail regarding the specific antibiotic(s) they used. This insufficiency in information provided also limited our ability to conduct sub-analyses on the classes of antibiotics. Another limitation of this study is the lack of information gathered regarding participants’ pre-conception antibiotic exposure. This may be important since the impact of antibiotics on the gastrointestinal microbiota can last for several months, if not longer [49]. Also, due to the voluntary nature of this study, it was not possible to ascertain causes of participant attrition and survey non-completion; however, there is a possibility that depression development may have contributed to one or both.

The recruited study cohort was relatively well-educated with a high socioeconomic status. This may have biased the results, especially towards a potentially lower incidence of ADSs. Moreover, the use of the word “wellbeing” in the study flyer may have attracted participants who were less likely to have had depression. Together, these limit the generalisability of the study’s findings. Although the initial target sample size was not met, this was not a major issue as the observed outcome incidence was almost three times the anticipated rate.

## 5. Conclusions

This study did not find a significant association between antibiotic exposure during pregnancy and the development of depressive symptoms. However, four factors identified previously to be significant independent predictors of ADSs were confirmed. The present study showed that experiencing ADSs is common, although it appears that many individuals with an elevated EPDS score do not receive a formal diagnosis of depression. Future research is warranted to investigate factors contributing to the apparent non-identification of individuals with high EPDS scores.

## Figures and Tables

**Table 1:** Basic demographic characteristics of participants and association with depressive symptoms (*n* = 977).

Variables	*n*	Without Depressive Symptoms (%)	With Depressive Symptoms (%)	*p*-Value	unadjOR (95% CI)
18–25 years of age 26–35 years of age 36 years and over	963	24 (3.0) 630 (78.4) 150 (18.7)	11 (6.9) 129 (81.1) 19 (11.9)	0.010	
Neither Aboriginal nor Torres Strait Islander Aboriginal or Torres Strait Islander	976	805 (98.8) 10 (1.2)	155 (96.3) 6 (3.7)	0.035	
Born in Australia Moved ≤ 10 years ago to Australia Moved > 10 years ago to Australia	975	678 (83.3) 53 (6.5) 83 (10.2)	128 (79.5) 16 (9.9) 17 (10.6)	0.305	
Married/Living with a partner Single/Separated/Divorced/In a relationship	976	791 (96.9) 25 (3.1)	150 (93.8) 10 (6.3)	0.061	
Household fortnightly income after tax < AUD 2000 Household fortnightly income after tax between AUD 2000 and AUD 5000 Household fortnightly income after tax > AUD 5000	940	70 (8.9) 515 (65.3) 204 (25.9)	27 (17.9) 109 (72.2) 15 (9.9)	<0.001	1.0 0.55 (0.34–0.90) 0.19 (0.10–0.38)
Other forms of education ^#^ University degree	977	169 (20.7) 647 (79.3)	57 (35.4) 104 (64.6)	<0.001	1.0 0.48 (0.33–0.69)
Unpaid job/study/etc. Paid job	977	96 (11.8) 720 (88.2)	37 (23.0) 124 (77.0)	<0.001	1.0 0.45 (0.29–0.68)

The totals do not always add up to 977 because of missing values. CI: confidence interval; EPDS: Edinburgh Postnatal Depression Scale; unadjOR: unadjusted odds ratio. ^#^ includes certificate/diploma, trade/apprenticeship, year 10 or year 12 or equivalent, no formal qualifications.

**Table 2:** Pregnancy-related characteristics and medical conditions and association with depressive symptoms (*n* = 977).

Variables	*n*	Without Depressive Symptoms (%)	With Depressive Symptoms (%)	*p*-Value	unadjOR (95% CI)
Unplanned pregnancy Planned pregnancy	967	131 (16.2) 678 (83.8)	45 (28.5) 113 (71.5)	<0.001	1.0 0.49 (0.33–0.72)
Singleton pregnancy Multiple pregnancy	975	805 (98.8) 10 (1.2)	157 (98.1) 3 (1.9)	0.458	
No gestational diabetes Gestational diabetes	963	699 (87.0) 104 (13.0)	133 (83.1) 27 (16.9)	0.206	
No high blood pressure disorders High blood pressure disorders	963	780 (97.1) 23 (2.9)	153 (95.6) 7 (4.4)	0.319	
No pain Pain	977	369 (45.2) 447(54.8)	65 (40.4) 96 (59.6)	0.261	
No iron deficiency, anaemia, or thalassaemia Iron deficiency, anaemia, or thalassaemia	963	781 (97.3) 22 (2.7)	154 (96.3) 6 (3.8)	0.445	
Able to eat (with or without nausea and/or vomiting) Nausea and vomiting causing an inability to eat	963	741 (92.3) 62 (7.7)	133 (83.1) 27 (16.9)	0.001	1.0 2.43 (1.49–3.95)
No antibiotic use Antibiotic use during pregnancy No probiotic use Probiotic use during pre-conception and pregnancy	970 971	663 (81.7) 149 (18.3) 627 (77.2) 185 (22.8)	125 (79.1) 33 (20.9) 124 (78.0) 35 (22.0)	0.504 0.837	

The totals do not always add up to 977 because of missing values. CI: confidence interval; EPDS: Edinburgh Postnatal Depression Scale; unadjOR: unadjusted odds ratio.

**Table 3:** Social and psychological factors prior to and during pregnancy and association with depressive symptoms (*n* = 977).

Variables	*n*	Without Depressive Symptoms (%)	With Depressive Symptoms (%)	*p*-Value	unadjOR (95% CI)
No personal history of depression Personal history of depression	977	629 (77.1) 187(22.9)	67 (41.6) 94 (58.4)	<0.001	1.0 4.72 (3.31–6.72)
No family history of mental health disorder(s) Family history of mental health disorder(s)	975	456 (56.0) 358 (44.0)	60 (37.3) 101 (62.7)	<0.001	1.0 2.14 (1.51–3.04)
Poor social support Moderate social support Strong social support	977	78 (9.6) 304 (37.3) 434 (53.2)	56 (34.8) 60 (37.3) 45 (28.0)	<0.001	1.0 0.28 (0.18–0.43) 0.14 (0.09–0.23)
No intimate partner emotional abuse within the prior 12 months	966	649 (80.5)	107 (66.9)	<0.001	1.0
Intimate partner emotional abuse within the prior 12 months		157 (19.5)	53 (33.1)		2.05 (1.41–2.97)
No intimate partner physical abuse within the prior 12 months	966	794 (98.5)	155 (96.9)	0.180	
Intimate partner physical abuse within the prior 12 months		12 (1.5)	5 (3.1)		

The totals do not always add up to 977 because of missing values. CI: confidence interval; EPDS: Edinburgh Postnatal Depression Scale; unadjOR: unadjusted odds ratio.

**Table 4:** Parity and lifestyle risk factors during pregnancy and association with depressive symptoms (*n* = 977).

Variables	*n*	Without Depressive Symptoms (%)	With Depressive Symptoms (%)	*p*-Value	unadjOR (95% CI)
Nulliparous individuals ^#^ Primiparous individuals Multiparous individuals	977	452 (55.4) 269 (33.0) 95 (11.6)	87 (54.0) 63 (39.1) 11 (6.8)	0.111	
Underweight pre-conception BMI Healthy weight pre-conception BMI Overweight pre-conception BMI Obese pre-conception BMI	966	22 (2.7) 380 (47.1) 232 (28.7) 173 (21.4)	2 (1.3) 61 (38.4) 46 (28.9) 50 (31.4)	0.033	
Non-cigarette smoker during pregnancy Occasionally/regularly smoked cigarettes during pregnancy ^&^	977	788 (96.6) 28 (3.4)	143 (88.8) 18 (11.2)	<0.001	1.0 3.54 (1.91–6.57)
No alcohol consumed during pregnancy Alcohol consumed during the pregnancy	977	703 (86.2) 113 (13.8)	143 (88.8) 18 (11.2)	0.381	
Not physically active during pregnancy Physically active during pregnancy	973	69 (8.5) 745 (91.5)	30 (18.9) 129 (81.1)	<0.001	1.0 0.40 (0.25–0.64)

The totals do not always up add to 977 because of missing values. ^#^ Nulliparous is defined as someone who has never given birth previously (regardless of outcome); Primiparous is defined as someone who has given birth once (including stillbirth (defined as miscarrying after 20 weeks)); Multiparous is defined as someone who has given birth more than once (including stillbirth). ^&^ This category includes those individuals who stopped smoking when they found out they were pregnant. BMI: body mass index; CI: confidence interval; EPDS: Edinburgh Postnatal Depression Scale; unadjOR: unadjusted odds ratio.

**Table 5:** Multiple logistic regression analysis results for predictors of developing depressive symptoms during pregnancy (*n* = 977).

Variables	adjOR (95% CI)	*p*-Value	adjOR (95% CI) Including Antibiotic	*p*-Value
Other forms of education ^#^ University degree	1.0 0.57 (0.38–0.84)	0.005	1.0 0.58 (0.39–0.86)	0.007
No personal history of depression Personal history of depression	1.0 4.21 (2.92–6.05)	<0.001	1.0 4.05 (2.81–5.85)	<0.001
No intimate partner emotional abuse within the prior 12 months Intimate partner emotional abuse within the prior 12 months	1.0 1.91 (1.28–2.84)	0.001	1.0 1.98 (1.33–2.95)	0.001
No or experienced nausea and/or vomiting able to eat Nausea and vomiting causing an inability to eat	1.0 1.91 (1.11–3.26)	0.019	1.0 1.96 (1.14–3.36)	0.014
No antibiotic use Antibiotic use during pregnancy			1.0 0.92 (0.58–1.46)	0.730

adjOR: adjusted odds ratio; CI: confidence interval; EPDS: Edinburgh Postnatal Depression Scale. ^#^ Other forms of education include certificate/diploma, trade/apprenticeship, year 10 or equivalent, year 12 or equivalent, no formal qualifications.

## Data Availability

The datasets presented in this article are not readily available because the data are part of an ongoing study. Requests to access the datasets should be directed to Corinne Mirkazemi (corinne.mirkazemi@utas.edu.au).

## Supplementary Materials

The following supporting information can be downloaded at: https://www.mdpi.com/article/10.3390/jcm13051434/s1; Table S1: Associations between antibiotic and probiotic use and developing depressive symptoms, Table S2: Sleep, anxiety, and stress levels, as well as experience of COVID-19 during the pregnancy, and association with depressive symptoms, Table S3: Inter-relationships between variables and the primary outcome (depressive symptoms), Table S4. Multiple logistic regression analysis results for predictors of developing depressive symptoms during pregnancy (sensitivity analysis) (*n* = 977).
