# Supplementary material for: Maternal Antibiotic Exposure and the Risk of Developing Antenatal Depressive Symptoms
Source: J Clin Med. 2024 Mar 1;13(5):1434. doi: 10.3390/jcm13051434 (PMC10932309; doi:10.3390/jcm13051434)
Supplement: Supplementary file 1 [file jcm-13-01434-s001.zip › jcm-2835587-supplementary.pdf]

Table S1. Associations between antibiotic and probiotic use and developing depressive symptoms (defined as having either an Edinburgh Postnatal Depression Scale of 13 or more and/or being diagnosed with depression) (n = 977).

**Variables (Total number)**	**n**	**Without depressive symptoms (%)**	**With depressive symptoms (%)**	***p*-Value**
No antibiotic use in 1st trimester	970	743 (91.5)	142 (89.9)	0.538
1st Trimester antibiotic use		69 (8.5)	16 (10.1)	
No antibiotic use in 2nd trimester	970	741 (91.3)	143 (90.5)	0.762
2ndTrimester antibiotic use		71 (8.7)	15 (9.5)	
No antibiotic use in 3rd trimester	970	785 (96.7)	152 (96.2)	0.764
3rd Trimester antibiotic use		27 (3.3)	6 (3.8)	
No probiotic use during pre-conception	971	729 (89.8)	148 (93.1)	0.241
Pre-conception probiotic use		83 (10.2)	11 (6.9)	
No probiotic use in 1st trimester	971	706 (86.9)	142 (89.3)	0.438
1st Trimester probiotic use		106 (13.1)	17 (10.7)	
No probiotic use in 2nd trimester	971	695 (85.6)	137 (86.2)	0.902
2nd Trimester probiotic use		117 (14.4)	22 (13.8)	
No probiotic use in 3rd trimester	971	689 (84.9)	135 (84.9)	1.000
3rd Trimester Probiotic use		123 (15.1)	24 (15.1)	

The totals do not always add up to 977 because of missing values.

Table S2. Sleep, anxiety, and stress levels, as well as experience of COVID-19 during the pregnancy, and association with depressive symptoms (defined as having either an Edinburgh Postnatal Depression Scale of 13 or more and/or being diagnosed with depression) (n = 977).

Variables	n	Without depressive symptoms (%)	With depressive symptoms (%)	*p*-Value
Poor quality of sleep	977	165 (20.2)	72 (44.7)	**<0.001**
Fair quality of sleep		367 (45.0)	63 (39.1)	
Good quality of sleep		284 (34.8)	26 (16.1)	
Normal + mild anxiety	977	750 (91.9)	72 (44.7)	**<0.001**
Moderate anxiety		52 (6.4)	46 (28.6)	
Severe + extremely severe anxiety		14 (1.7)	43 (26.7)	
Normal + mild Stress	977	783 (96.0)	96 (59.6)	**<0.001**
Moderate Stress		27 (3.3)	25 (15.5)	
Severe + extremely severe Stress		6 (0.7)	40 (24.8)	
Had not been diagnosed with COVID-19	977	621 (76.1)	117 (72.7)	0.367
Diagnosed with COVID-19		195 (23.9)	44 (27.3)	
Close contact diagnosed with	977			
COVID-19		686 (84.1)	134 (83.2)	0.814
Close contact not diagnosed with COVID-19				
		130 (15.9)	27 (16.8)	
Pregnancy not affected by COVID-19	977	259 (31.7)	36 (22.4)	0.019
Pregnancy affected by COVID-19		557 (68.3)	125 (77.6)	
Negative effect of social isolation associated with COVID-19 on mental health	975			
None to a little				
Moderate		436 (53.6)	35 (21.7)	**<0.001**
A lot		219 (26.9)	55 (34.2)	
		159 (19.5)	71 (44.1)	
Negative effect of COVID-19 on mental health	977			
None to a little				
Moderate		358 (43.9)	24 (14.9)	**<0.001**
A lot		287 (35.2)	57 (35.4)	
		171 (21.0)	80 (49.7)	

The totals do not always add up to 977 because of missing values.

Table S3. Inter-relationships between variables and the primary outcome (depressive symptoms). Those analyses with $p < 0.001$ were deemed to be significantly related and are shown in bold.

Independent variables	Age	Income	Education	Employment *	Px Hx of depression	Planned pregnancy	Cigarette smoking	Physical activity	N and V	Fx Hx of mental health disorder#	Social support	Abuse&
**Age**	***											
**Income**	0.011	***										
**Education**	**<0.001**	**<0.001**	***									
**Employment***	0.001	**<0.001**	**<0.001**	***								
**Px Hx of depression**	0.002	**<0.001**	0.003	**<0.001**	***							
**Planned pregnancy**	0.009	**<0.001**	**<0.001**	**<0.001**	0.089	***						
**Cigarette smoking**	0.166	0.001	**<0.001**	0.024	0.002	0.002	***					
**Physical activity**	0.048	0.016	**<0.001**	0.002	**<0.001**	0.56	**<0.001**	***				
**N and V**	0.124	0.868	0.034	0.072	0.003	0.312	0.599	0.005	***			
**Fx Hx of mental health disorder#**	0.024	0.044	0.149	0.040	**<0.001**	0.933	0.006	0.202	0.824	***		
**Social support**	0.098	**<0.001**	**<0.001**	**<0.001**	**<0.001**	**<0.001**	0.009	**<0.001**	0.494	0.436	***	
**Abuse &**	0.570	0.245	1.000	0.366	0.001	**<0.001**	1.000	0.364	1.000	0.004	0.003	***

Fx: Family; Hx: history; N and V: Nausea and vomiting; Px: personal. * paid vs. unpaid job. # including family history of depression. & abuse= Intimate partner emotional abuse.

Table S4. Multiple logistic regression analysis results for predictors of developing depressive symptoms during pregnancy (sensitivity analysis) (N = 977).

**Variables**	**n**	**unadjOR (95%CI)**	***p*-Value**	**adjOR (95%CI)**	***p*-Value**	**adjOR (95%CI) including antibiotic**	***p*-Value**
Other forms of education [#]	977	**1.0**	**<0.001**	**1.0**	**0.006**	**1.0**	**0.009**
University degree		**0.46 (0.31-0.68)**		**0.55 (0.36-0.84)**		**0.57 (0.37-0.87)**	
No personal history of depression	977	**1.0**	**<0.001**	**1.0**	**<0.001**	**1.0**	**<0.001**
Personal history of depression		**4.00 (2.76-5.81)**		**3.46 (2.34-5.12)**		**3.29 (2.12-4.89)**	
No intimate partner emotional abuse within the prior 12 months	966	**1.0**	**<0.001**	**1.0**	**0.002**	**1.0**	**0.001**
Intimate partner emotional abuse within the prior 12 months		**2.16 (1.46-3.20)**		**1.93 (1.27-2.94)**		**2.02 (1.32-3.08)**	
Able to eat (with or without nausea and/or vomiting)	963	**1.0**	**<0.001**	**1.0**	**0.029**	**1.0**	**0.023**
Nausea and vomiting causing an inability to eat		**2.51 (1.51-4.18)**		**1.88 (1.06-3.31)**		**1.94 (1.10-3.42)**	
18-25 years of age	963	**1.0**	**0.009**	1.0	0.215	1.0	0.166
26-35 years of age		**0.42 (0.20-0.91)**		0.74 (0.32-1.72)		0.72 (0.31-1.67)	
36 years and over		**0.24 (0.10-0.60)**		0.47 (0.17-1.25)		0.43 (0.16-1.16)	
No antibiotic use	970	1.0	0.359			1.0	0.811
Antibiotic use during pregnancy		1.23 (0.79-1.93)				0.94 (0.58-1.53)	

The totals do not always add up to 977 because of missing values. adjOR: adjusted odds ratio; CI: confidence interval; unadjOR: unadjusted odds ratio. [#] includes certificate/diploma, trade/apprenticeship, year 10 or year 12 or equivalent, no formal qualifications.
